# Novel plasmid curing mediated restoration of antimicrobial sensitivity by *Nigella sativa* extract against multidrug resistant *Staphylococcus aureus*

**DOI:** 10.1038/s41598-025-33667-3

**Published:** 2026-01-18

**Authors:** Adel Attia M. Ahmad, Ashraf M. O. Abdelwahab, Esraa Fawzy, Tarek Khamis, Mohamed Abdelmoneim, Marwa I. Abd El-Hamid

**Affiliations:** 1 Department of Microbiology, Faculty of Veterinary Medicine, Zagazig University, Zagazig, Egypt; 2 Department of Pharmacology, Faculty of Veterinary Medicine, Zagazig University, Zagazig, Egypt

**Keywords:** MDR *S. aureus*, Plasmid curing, *Nigella sativa*, Thymoquinone, Resistant phenotype variants, Plasmid curing, SDS, Drug discovery, Microbiology

## Abstract

Plasmid-mediated antimicrobial resistance is a major contributor to infections associated with high morbidity and mortality. Given the urgent demand for effective and safe elimination of plasmids from multidrug-resistant (MDR), this study is the first to examine the potential of *Nigella sativa* essential oil (NSO) and thymoquinone (TQ) in eliminating antimicrobial resistance plasmid from *Staphylococcus aureus*. Plasmid profiles of resistant variants derived from the original MDR strains following exposure to sublethal concentrations of NSO and TQ were analyzed and compared with those of the untreated parent strains using agar gel electrophoresis. Computational analyses were performed to evaluate the interactions between eleven phytoconstituents of *Nigella sativa* constituents and cytidylyltransferase (CTP) and glutamine amidotransferase (Gat-D) enzymes, which are involved in cell wall biosynthesis. NSO and TQ exhibited plasmid- curing and MDR reversal effects comparable to SDS. Remarkably, their combination with antimicrobials or SDS resulted in superior plasmid elimination efficiency. The eleven NSO constituents demonstrated moderate to strong binding affinities with CTP and Gat-D enzymes, thereby impairing their catalytic function by obstructing substrate interaction. The plasmid curing property offers a novel therapeutic strategy for combating antibiotic resistance by eliminating resistance genes from infectious *S. aureus*.

**Supplementary Information:**

The online version contains supplementary material available at 10.1038/s41598-025-33667-3.

## Introduction

Plasmids, transposons, pathogenicity islands, insertion sequences, and chromosomal cassettes are mobile genetic elements (MGEs) that facilitate the transfer of antimicrobial resistance markers and virulence traits in bacterial populations^1–3^. To control bacterial infections, eliminating plasmids that carry antibiotic resistance genes is essential. Lateral plasmid transfer via sexual contact, known as bacterial conjugation or through asexual means such as plasmid transformation are the most significant challenges in the management of infectious diseases. Plasmids facilitate the transfer of resistance genes to antibiotics, heavy metals, and biocides, along with encoding genes for virulence factors, host survival mechanisms, and toxins^4^. The acquisition of these genes via a transferred plasmid into *S. aureus* beautifies the organism’s adaptability and proliferation in its surrounding environments due to the co-selective advantage conferred^5^. Lateral antibiotic resistance transfer between bacterial species is inhibited by plasmid curing, which removes plasmids from bacterial cells. Antimicrobial resistance caused by plasmids in the rich gastrointestinal microbiome of humans and animals has drawn attention. Various compounds derived from plants have shown the ability to exclude plasmids out of MDR *Staphylococcus aureus*, offering a viable alternative to traditional agents such as acridine orange and sodium dodecyl sulfate (SDS). Extracts from *Alcea arebellensis*, *Alpinia galangal*, *Plumbago*, and fermented olive leaves have shown efficacy in removing resistance plasmids and restoring antibiotic sensitivity^6–8^. These plant-derived compounds often act through conjugation inhibition or by direct plasmid destabilization^9^. Compared to SDS which, despite its effectiveness, poses cytotoxic and mutagenic risks, phytochemicals are generally safer and more biocompatible, making them suitable for therapeutic exploration^10–12^. However, these agents face limitations such as inconsistent efficacy, poor bioavailability, and lake of standardized extraction method^13^. While some compounds like curcumin and thymol exhibit potent antibiofilm and plasmid-curing effects, their application is limited due to insufficient pharmacokinetic data and lack of regulatory approval^14,15^. To date, no plant-based plasmid-curing agent has received formal clinical approval for use against *S. aureus*, although several agents are under investigation as adjunct therapies^16^. In vitro studies suggest that plant extracts may offer comparable or superior efficacy to SDS, especially when optimized through nanotechnology or advanced formulation techniques^4^. While natural and plant-based plasmid-curing agents are being actively investigated, most currently available options lack clinical effectiveness. There is a pressing need to discover safe, plant-derived antibacterial compounds that can serve as viable alternatives to synthetic drugs. Ideal candidates should exhibit robust mechanisms of action across diverse bacterial growth conditions, effectively target a wide range of plasmids and bacterial strains, and preferably inhibit bacterial conjugation^17,18^. One of the most promising clinical strategies to curb the spread of resistance and virulence factors involves using plant-based compounds to block the transfer of conjugative plasmids between both related and unrelated bacterial species. *Nigella sativa*, commonly known as black seed, is a medicinal plant with a long history of use in traditional Islamic medicine, where it is regarded as a remedy for various ailments; its widespread use is prominent in the Middle East, South Asia, and Africa^19^. Modern pharmacological studies have confirmed its safety and biocompatibility with thymoquinone. Its principal bioactive compound exhibits antioxidant, anti-inflammatory, and antimicrobial properties^20,21^. *Nigella sativa* demonstrates potent antibacterial activity against MDR *S. aureus*, including methicillin-resistant strains (MRSA), by disrupting cell envelope integrity and increasing membrane permeability, leading to cell lysis^22,23^. It also inhibits biofilm formation and generates reactive oxygen species (ROS), contributing to its bactericidal effects^24,25^. *Nigella sativa* oil extract inhibits bacterial conjugation by interfering with plasmid transfer machinery, notably blocking vancomycin resistance gene transfer from *Enterococcus faecium* to *S. aureus*. This effect is dose-dependent and linked to suppression of transposon-mediated gene mobility, offering a natural strategy to curb horizontal resistance spread^17^. *Nigella sativa* exerts antibacterial effects on *S. aureus* by targeting its cell envelope through multiple mechanisms. Its essential oil, particularly thymoquinone, disrupts membrane integrity, increases permeability, and induces leakage of intracellular contents, leading to cell lysis^26^. Studies have shown that *Nigella sativa* downregulates key cell wall biosynthesis genes such as murF and pbp2, which are essential for peptidoglycan synthesis and penicillin-binding protein function, respectively^27^. This genetic suppression compromises the integrity of cell wall structure and enhances susceptibility to antibiotics. Sublethal exposure to *Nigella sativa* extract may compromise bacterial cell wall integrity, which in turn may destabilize plasmid maintenance mechanisms. This interplay suggests that cell envelope stress can indirectly affect plasmid stability. Antibiotic resistance mechanisms are dependent on the complicated structure of the bacterial cell wall. Amidated peptidoglycan plays an important role in the complex architecture of the cell wall. Gat-D, in complex with the MurT, catalyzes the amidation reaction in the cell wall peptidoglycan biosynthetic pathway; ammonia produced by Gat-D amidates the stem peptide residue D-iso-Glu of lipid II to D-iso-Gln in the amidated lipid II. The MurT-GatD complex is required for cell viability, full resistance to β-lactam antibiotics, and resistance to human lysozyme and is recognized as an attractive target for new antimicrobials^28^. Glycerol-3-phosphate cytidylyltransferase (CTP) catalyzes the formation of CDP-glycerol, which covalently couples the teichoic acid to the peptidoglycan polymer in the cell wall of Gram-positive bacteria^29^. Teichoic acids and their attachment to peptidoglycan contribute to bacterial cell surface charge and hydrophobicity that affect the binding of extracellular molecules^30^. This contributes to pathogenicity, host interaction, biofilm development, and antibiotic resistance. Because bacterial CTP: Glycerol-3-phosphate cytidylyltransferases are crucial for cell wall manufacture, they may be targets for new antibiotics. The primary target of *N. sativa* against *S. aureus* is the cell envelope, and this impact also affects plasmids that are passed on to subsequent generations, a factor previously overlooked. The study focuses on the dynamics of *N. sativa* active components in the progeny of the exposed parental isolates.

## Results

### Antimicrobial resistance patterns of *S. aureus* isolates

Eleven *S. aureus* isolates from urine samples were resistant to nine or more of the tested antimicrobials (Table 1). Moreover, nine *S. aureus* isolates from pus samples showed resistance to at least ten antimicrobials. Notably, all isolates from both urine and pus were resistant to ampicillin and amoxycillin/clavulanic acid. Among these strains, tazobactam emerged as the most effective antimicrobial agent. Additionally, nine *S. aureus* strains isolated from the milk of cows suffering from mastitis exhibited resistance to three or more of the tested antimicrobials (Table 2). All strains from this source were resistant to ampicillin and lincomycin, while each strain demonstrated sensitivity to both ofloxacin and linezolid (Supplementary Figure S1).

When compared to the *S. aureus* isolated from human urine and pus samples, those from mastitis milk displayed a lower number of resistance patterns. Statistical analysis showed significant differences in the antimicrobial resistance patterns of tested *S. aureus* isolates from human and mastitis cows’ milk samples to amoxycillin/clavulanic acid (*p* < 0.0001), ampicillin/sulbactam, gentamicin and ofloxacin antimicrobials (*p* = 0.005, 0.001, and 0.001, respectively) (Table 3).

**Table 1:** Antimicrobial resistance patterns of *S. aureus* strains isolated from human samples and their susceptibility to thymoquinone.

Strain code No.	Antimicrobial resistance pattern																NO. of drugs to which the strain was resistant	Thymo-quinone	
	AMC	AMP	FEP	L	AZM	CN	CTX	DA	TOB	SAM	OFX	DO	SXT	C	LNZ	TPZ		IZD (mm)	MIC µg/mL
UR1	r	r	r	r	r	r	r	r	r	s	r	r	s	s	s	s	11	34	12
UR2	r	r	r	r	r	r	r	r	r	r	r	s	r	r	r	s	14	42	12
UR3	r	r	r	r	r	r	r	r	s	s	r	r	r	r	s	r	13	34	6
UR4	r	r	r	r	r	r	r	r	r	r	r	s	r	s	s	s	12	40	4
UR5	r	r	r	r	r	r	r	r	r	r	s	s	s	r	s	s	11	30	1
UR6	r	r	r	r	r	r	r	r	s	r	r	r	r	r	r	r	15	6	4
UR7	r	r	r	s	r	r	r	s	r	r	r	r	r	s	s	s	11	6	2
UR8	r	r	r	r	s	r	r	r	r	r	s	s	s	s	s	r	10	18	0
UR9	r	r	r	r	r	s	s	r	s	s	s	r	r	r	s	s	9	35	2
UR10	r	r	r	r	r	r	s	r	s	r	r	r	r	r	r	s	13	11	3
UR11	r	r	r	r	r	r	s	r	s	r	r	r	r	r	r	s	13	11	3
PU1	r	r	r	r	r	r	r	r	r	s	r	r	s	s	s	s	11	14	0
PU2	r	r	r	r	r	r	r	r	r	r	s	s	s	s	s	r	11	25	4
PU3	r	r	r	r	r	r	r	r	r	r	r	r	s	s	r	s	13	50	10
PU4	r	r	r	r	r	r	r	r	r	r	r	r	s	s	r	r	14	46	9
PU5	r	r	r	r	r	r	r	r	s	r	s	r	r	r	r	s	13	8	2
PU6	r	r	r	r	r	r	r	r	r	r	r	r	r	s	s	r	14	6	0
PU7	r	r	r	r	r	r	r	s	r	s	r	s	r	s	s	s	10	35	12
PU8	r	r	r	r	r	r	r	r	s	r	r	r	r	r	r	s	14	23	5
PU9	r	r	s	r	r	r	r	r	r	s	s	r	r	r	r	s	12	20	7
Resistance percentage	100	100	95	95	95	95	85	90	65	70	70	70	65	50	45	30			

*UR* urine, *PU* pus, r resistant, *AMC* amoxycillin/clavulanic acid, *AMP* ampicillin, *FEP* cefepime, *L* lincomycin, *AZM*: azithromycin, *CN* gentamicin, *CTX* cefotaxime, *DA* clindamycin, *TOB* tobramycin, *SAM* ampicillin/sulbactam, *OFX* ofloxacin, *DO* doxycycline, *SXT* trimethoprim/sulphamethoxazol, *C* chloramphenicol, *LZD* linezolid, *TPZ* tazobactam, *IZD* inhibition zone diameter, *MIC* minimal inhibitory concentration, *r* resistant, *s* sensitive. Underlined strains were used for subsequent plasmid curing.

**Table 2:** Antimicrobial resistance patterns of *S. aureus* strains isolated from mastitis cows’ milk and their susceptibility to thymoquinone.

Strain code No.	Antimicrobial resistance pattern														NO. of drugs to which the strain was resistant	Thymo-quinone	
	L	AMP	FEP	CTX	AZM	DA	DO	C	AMC	SXT	TOB	CN	TPZ	SAM		IZD (mm)	MIC µg/mL
MK1	r	r	r	s	r	r	s	r	r	s	s	s	r	s	8	50	12
MK2	r	r	r	s	s	s	s	s	s	s	s	s	s	s	3	46	9
MK3	r	r	r	r	s	s	s	s	r	s	s	s	s	s	5	40	9
MK4	r	r	r	r	r	r	r	s	s	s	s	s	s	s	7	36	12
MK5	r	r	r	r	r	s	r	s	r	r	r	r	s	r	11	46	8
MK6	r	r	r	r	r	r	s	r	s	s	s	r	s	s	8	42	8
MK7	r	r	r	r	r	r	r	r	s	r	r	s	s	s	10	22	6
MK8	r	r	s	r	r	r	r	s	s	r	r	s	s	s	8	30	7
MK9	r	r	r	r	r	r	s	r	s	s	s	r	s	s	8	23	7
Resistance percentage	100	100	88.9	77.8		66.7	44.4		33.3				11.1				

*MK* milk, *r* resistant, *L* lincomycin, *AMP* ampicillin, *FEP* cefepime, *CTX* cefotaxime, *AZM* azithromycin, *DA* clindamycin, *DO* doxycycline, *C* chloramphenicol, *AMC* amoxycillin/clavulanic acid, *SXT* trimethoprim/sulphamethoxazol, *TOB* tobramycin, *CN* gentamicin, *TPZ* tazobactam, *SAM* ampicillin/sulbactam, *IZD* inhibition zone diameter, *MIC* minimal inhibitory concentration, *r* resistant, *s* sensitive. Underlined strains were used for subsequent plasmid curing. All strains were sensitive to linezolid and ofloxacin.

**Table 3:** Antimicrobial resistance patterns of *S. aureus* strains isolated from human and mastitis cows’ milk samples.

Antimicrobial category	Antimicrobial agent	No. of *S. aureus* isolates (%)		*p*-value	Total no. of *S. aureus* isolates (%) (*n* = 29)
		Human (*n* = 20)	Cattle (*n* = 9)		
β-lactam combination agents	AMC	20 (100)	3 (33.3)	< 0.0001***	23 (79.3)
	SAM	14 (70)	1 (11.1)	0.005**	15 (51.7)
	TPZ	6 (30)	1 (11.1)	0.382	7 (24.1)
Penicillins	AMP	20 (100)	9 (100)	NA	29 (100)
Cephems	FEP	19 (95)	8 (88.9)	1	27 (93.1)
	CTX	17 (85)	7 (77.8)	1	24 (82.8)
Lincosamides	L	19 (95)	9 (100)	1	28 (96.6)
	DA	18 (90)	6 (66.7)	0.287	24 (82.8)
Macrolides	AZM	19 (95)	7 (77.8)	0.22	26 (89.7)
Aminoglycoside*s*	CN	19 (95)	3 (33.3)	0.001**	22 (75.9)
	TOB	13 (65)	3 (33.3)	0.226	16 (55.2)
Fluoroquinolones	OFX	14 (70)	0	0.001**	14 (48.3)
Tetracyclines	DO	14 (70)	4 (44.4)	0.237	18 (62.1)
Folate pathway antagonists	SXT	13 (65)	3 (33.3)	0.226	16 (55.2)
Phenicols	C	10 (50)	4 (44.4)	1	14 (48.3)
Oxazolidinones	LZD	9 (45)	0	0.027*	9 (31)
TQ		11 (55)	9 (100)	0.027*	20 (69)

*AMC* amoxycillin/clavulanic acid, *AMP* ampicillin, *FEP* cefepime, *L* lincomycin, *AZM* azithromycin, *CN* gentamicin, *CTX* cefotaxime, *DA* clindamycin, *TOB* tobramycin, *SAM* ampicillin/sulbactam, *OFX* ofloxacin, *DO* doxycycline, *SXT* trimethoprim/sulphamethoxazole, *C* chloramphenicol, *LZD* linezolid, *TPZ* tazobactam, *TQ* Thymoquinone. *NA* non-applicable; *p < 0.05; **p < 0.01, ***p < 0.001.

### Effectiveness of plasmid curing in resistant phenotype variants of *S. aureus* treated with *N. sativa* essential oil

**Fig. 1:**
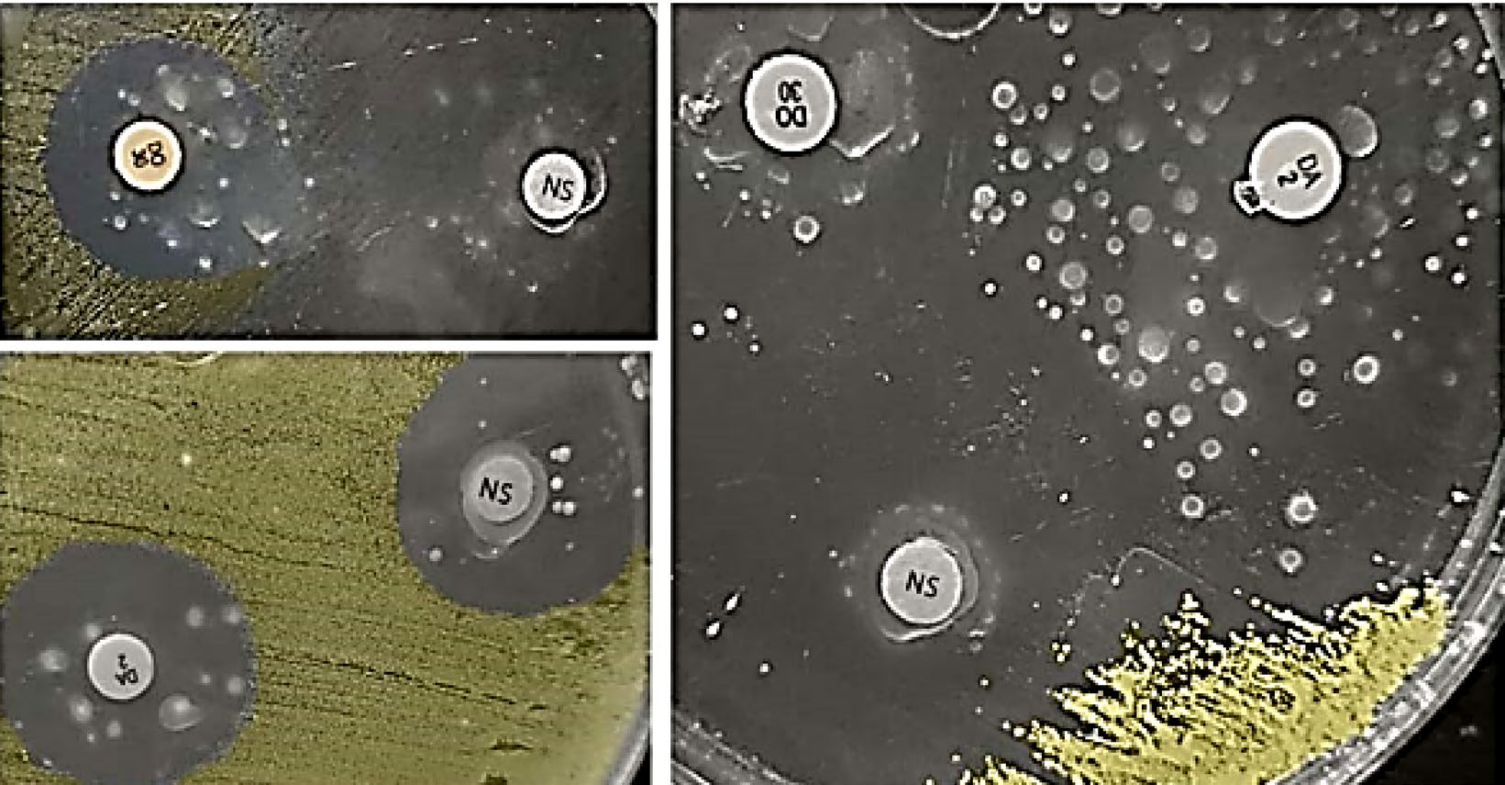
Parent *S. aureus* isolates give rise to resistant phenotypes against *Nigella sativa* (NS) essential oil and/or antimicrobials. Resistance to NS was evidenced by colony growth within the inhibition zones of NS discs (20 µL/disc). To assess combined resistance, bacteria were first exposed to NS and after 30 min, NS disc removal and antimicrobial disc placement were done. Regardless of whether resistance developed against NS alone or the NS/antimicrobial combinations (DO and DA), the resulting colonies displayed unusual traits; they were transparent, had intact margins, varied in size, and lacked their typical pigmentation.

Plasmid profiles of resistant phenotype variants (RPVs) obtained after NSO treatment (NSO-RPVs) were compared with those of the untreated parent strains (MK4, MK6, UR1, UR9, PU1, and PU9) as shown in Supplementary Table T1. These strains were selected based on the presence of plasmid-encoded antibiotic resistance markers. The RPV exhibited characteristics such as being mucoid, small, round, circumscribed, and non-pigmented, as illustrated in Fig. 1. The non-treated parent strains had between two to five plasmids, with sizes ranging from 6.48 to 51.36 kb, indicating a multi-plasmidic nature with a total of nine distinct molecular weight plasmids. All parent strains, except for one, contained at least one small plasmid measuring less than 10 kb. In contrast, the NSO-PRV showed a loss of one to three plasmids per strain, regardless of the plasmid size, as summarized in Supplementary Table T1. Out of the 19 analyzed plasmids, 12 (63.2%) were successfully cured or lost following exposure to NSO alone. The combinations of NSO/DO, NSO/AZM, and NSO/OFX demonstrated exceptional plasmid eradication efficacy achieving clearance rates of 89.5% (17/19), 78.9%, (15/19) and 73.7% (14/19), respectively and indicating their effectiveness as treatment options.

### Effectiveness of plasmid curing in resistant phenotype variants of *S. aureus* treated with TQ

**Fig. 2:**
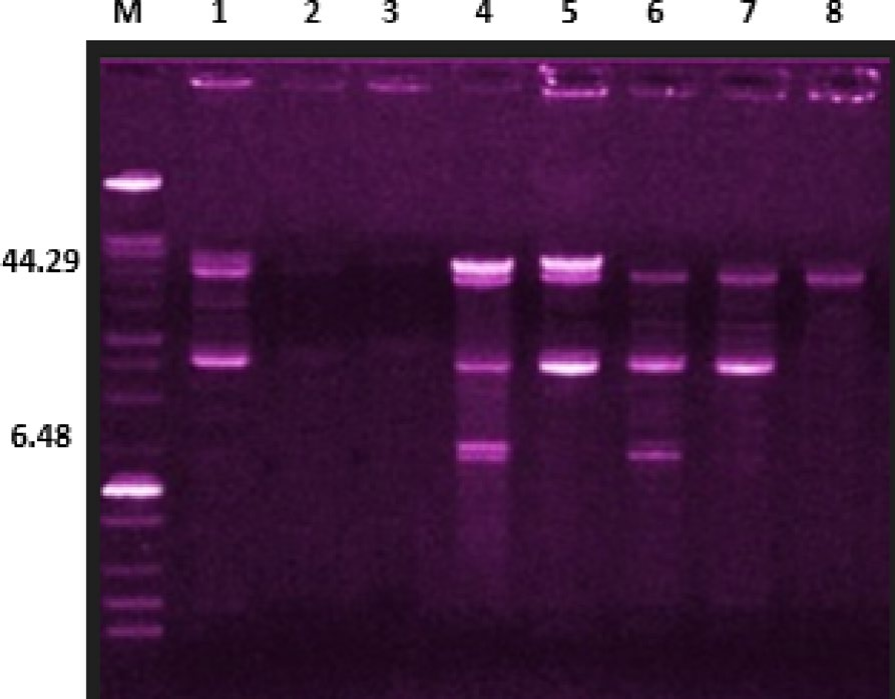
Plasmid profiles of *S. aureus* parent isolates and their resistant variants subjected to thymoquinone (TQ) and/or antimicrobials and sodium dodecyl sulfate (SDS) and/or TQ. Lanes 1 and 4: parent isolates from pus (PU) samples; PU1 and PU9, respectively, lanes 2 and 3: curing results of PU1 with TQ and SDS, respectively, lanes 5, 6, 7 and 8: curing results of PU9 with SDS, TQ, TQ/doxycycline and TQ/doxycycline/gentamycin, respectively, M: 100 Kb marker.

The plasmid profiles of RPV subjected to 0.5MIC-TQ (TQ-RPV), those exposed to TQ/drug combinations, SDS, and SDS/0.5MIC-TQ combinations were analyzed and compared to those of the non-treated parent strains (MK4, MK6, UR1, UR9, PU1, PU9) (Fig. 2 and Supplementary Table T2). The TQ-RPV exhibited a loss of one to four plasmids per strain, regardless of their size in the strains examined. A total of 8 out of 19 plasmids (42.1%) were successfully cured among the TQ-RPV. The observed variations in plasmid curing percentages across different treatments highlight the differential efficacy of NSO, TQ, and their combinations. NSO and TQ demonstrated comparable curing effects with mean percentages of 58 and 57.3%, respectively with significant differences between them except in isolate MK4. When compared to SDS, NSO showed a slightly lower curing rate. Although SDS achieved higher mean percentage (66.4%), the difference remained statistically insignificant. Notably, the combination treatments NSO/DO and TQ/DO + CN yielded the significant (*p* < 0.0001) plasmid curing rate (92.5% each) suggesting a strong synergistic interaction with doxycycline. These findings support the potential of NSO and TQ, particularly in combination with conventional antibiotics, to enhance plasmid elimination and possibly reduce resistance in *S. aureus* strains.

### Reversion of the cured antibiotic-resistant phenotypes to the sensitive category

**Fig. 3:**
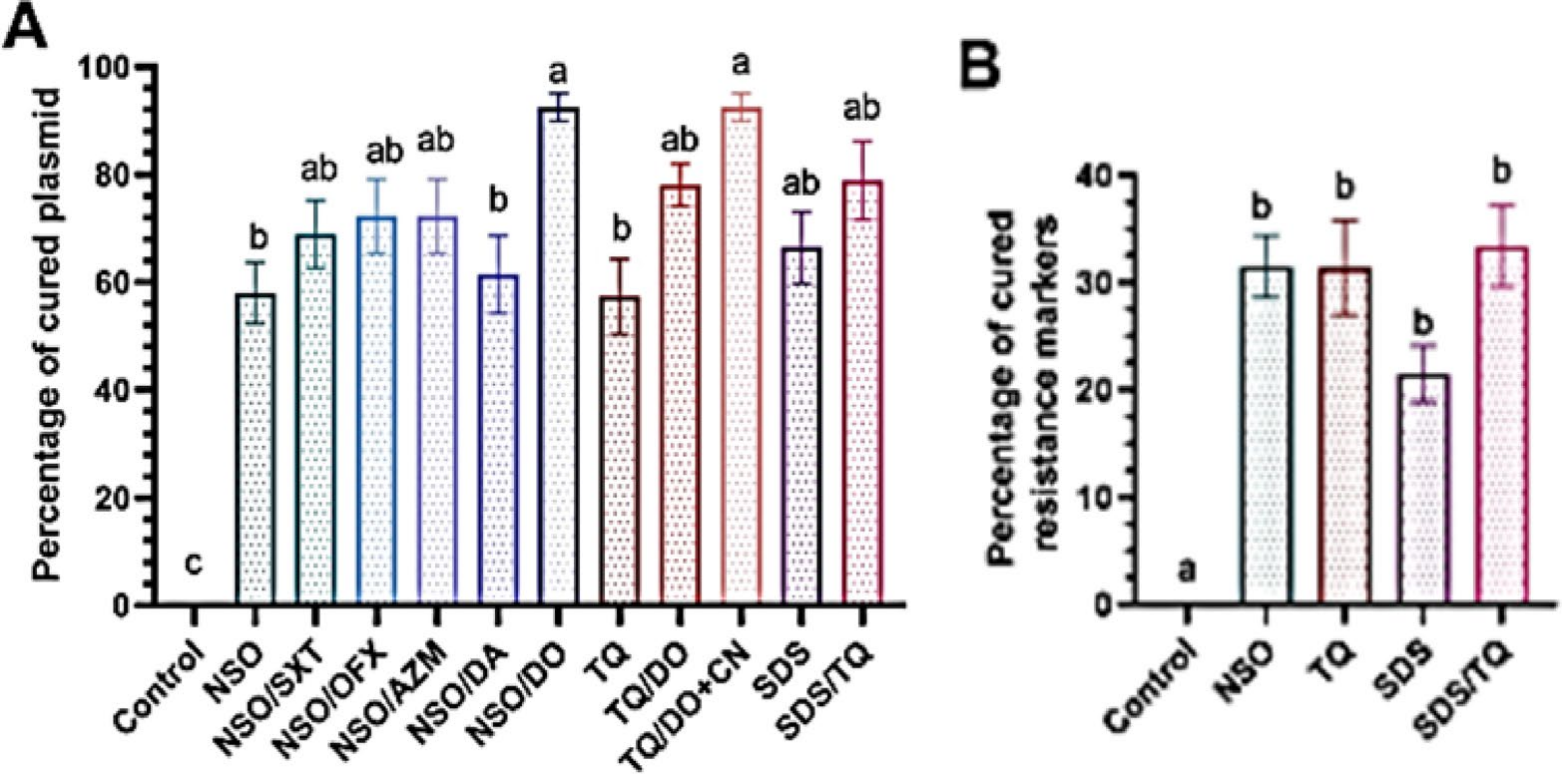
Percentages of cured plasmids in *Staphylococcus aureus* strains treated with *Nigella sativa* oil (NSO), thymoquinone (TQ) and their combinations with antimicrobials (**A**) along with the percentages of cured resistance markers. (**B**) Results were expressed as the mean of percentages of cured plasmids calculated as: number of plasmids in treated strain/number of plasmids in untreated strain) × 100 basing on triplicate experiments for each of the six tested *S. aureus* strains with standard error of the mean (SEM) shown as error bars. Means with different superscript letters (a–c) indicate statistically significant differences at *p* < 0.05. *AZM* azithromycin, *CN* gentamicin, *OFX* ofloxacin, *DO* doxycycline, *SXT* trimethoprim/sulfamethoxazole, *SDS* sodium dodecyl sulfate.

The investigation focused on the elimination of antibiotic resistance genes in the cured resistant phenotypes. Subsequent to subculturing on a medium devoid of antibiotics, the cured phenotypes developed into typical golden yellow pigmented colonies. The parent strains that underwent treatment produced cured resistant phenotypes that became susceptible to one or more antibiotics to which the original strain had previously exhibited resistance (Supplementary Tables T1 and T2). Following plasmid extraction, the resistant variants treated with NSO and TQ exhibited sensitivity to one to four antimicrobials. After treatment, all strains affected by NSO lost their antibiotic resistance indicators with a range of one to three observed across different strains. The plasmid removal process facilitated by NSO resulted in the elimination of antibiotic resistance markers from FEP, DO, AZM, SXT, CN, DA and OFX (Supplementary Table T1 and Fig. 3). Similarly, the removal of plasmids using 0.5XMIC-TQ in treated strains resulted in the loss of antibiotic resistance markers such as AZM, TOB, DO, DA, OFX and CN (Supplementary Table T2 and Fig. 3). Notably, five out of six strains (83.3%) exhibited a loss of antibiotic resistance markers after treatment with TQ. In contrast, all six strains (100%) lost antibiotic resistance markers after treatment with SDS.

### Molecular docking conformations and interactions between *N. sativa* components and cell wall enzymes

#### Glutamine amidotransferase

**Fig. 4:**
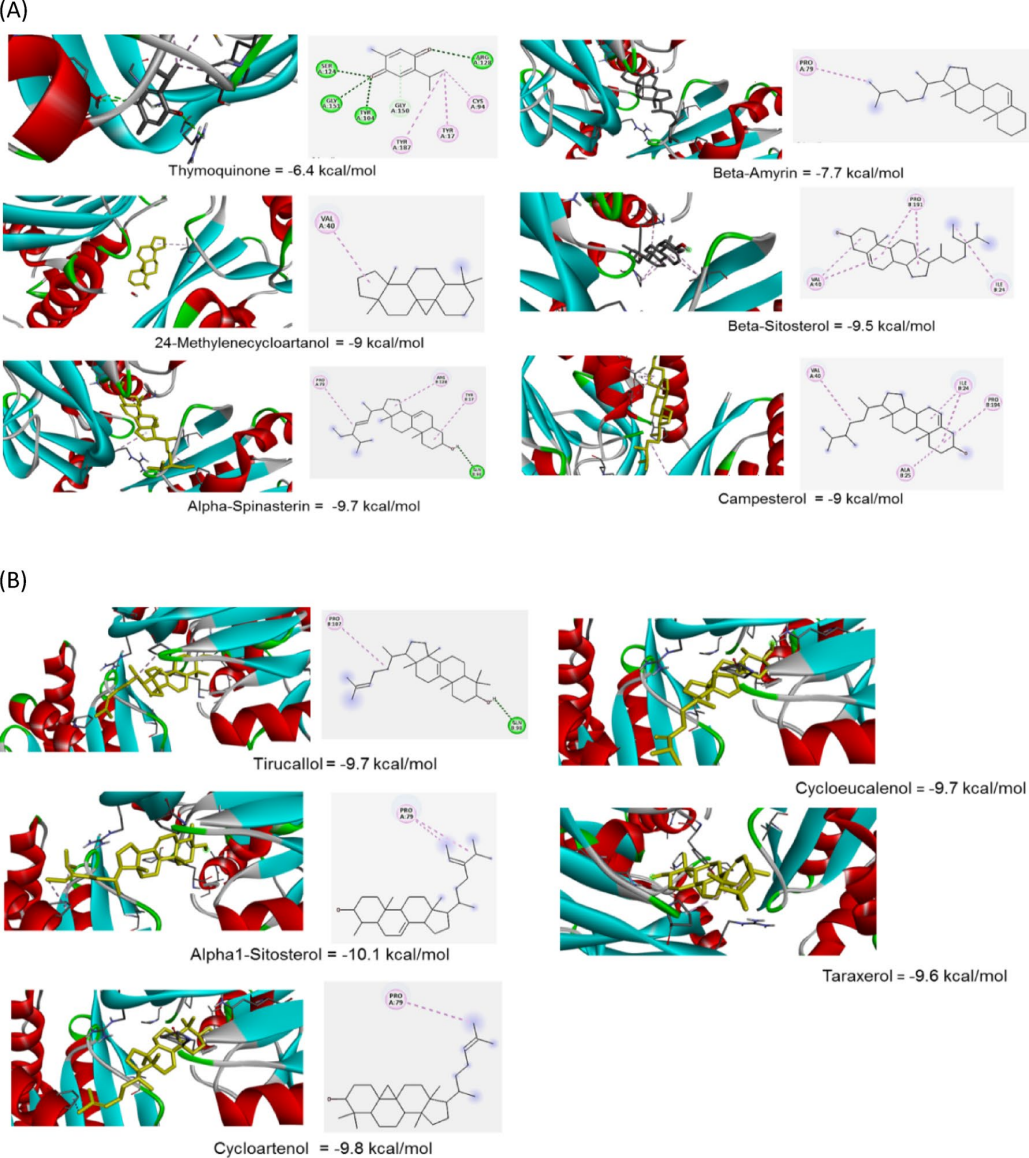
(**A**)Three-dimensional representation and 2D stick model amino acids of glutamine amidotransferase (Gat-D) bonding with six *N. sativa* active components. Key colors in 2D-dimension representation: light blue: Van der Waals, green: conventional hydrogen bonding, orange: Hydrophobic interactions, pink: Pi-Pi stacked, red: unfavorable acceptor and violet: Pi-sigma. (**B**) Three-dimension representation and 2D stick model amino acids of glutamine amidotransferase bonding with five *N. sativa* active components. Key colors in 2D-dimension representation: light blue: Van der Waals, green: conventional hydrogen bonding, orange: Hydrophobic interactions, pink: Pi-Pi stacked, red: unfavorable acceptor and violet: Pi-sigma.

Thymoquinone bound to glutamine amidotransferase with a moderate binding affinity (−6.4 Kcal/moL). Conventional hydrogen bonding of TQ with SER124, GLY151, TYR104, GLY150, and ARG128 stabilized positioning within the enzyme’s active site and altered the enzyme’s conformation in a way that reduced its ability to catalyze the reaction (Fig. 4A, Supplementary Table T3). The alkyl and π-alkyl interactions helped in stabilizing the ligand inside, block access to the enzyme’s substrate binding site, and enhanced the overall strength of the binding. The inhibition might be non-competitive, which disrupted the enzyme’s function even when the substrate was bound. The combination of hydrogen bonds and Van der Waals interactions between thymoquinone and glutamine amidotransferase likely led to strong binding, which prevented the enzyme from binding its natural substrate and disrupted catalytic activity. This strong and stable binding could be a key factor in thymoquinone’s role as an effective enzyme inhibitor. Alpha-spinasterin and tirucallol bound to Gat-D through a combination of alkyl (hydrophobic) interactions and a conventional hydrogen bond (Fig. 4A,B). This interaction is likely driven by both the hydrophobic nature and the formation of a specific hydrogen bond within the enzyme’s binding pocket. Beta-armyrin, 24-Methylene-cycloartanol, cycloartenol, campesterol, alpha1-sitosterol, and beta-sitosterol displayed binding affinities that were moderately strong (Fig. 4A,B and Supplementary Table T3). Even without typical hydrogen bonds, these ligands, which interacted primarily through hydrophobic forces, likely resided within a water-repelling region close to the enzyme’s active site. This positioning could hinder enzyme function by preventing substrate binding, triggering alterations in the enzyme’s shape, or affecting its activity at a distant regulatory site.

#### Cytidylyltransferase

**Fig. 5:**
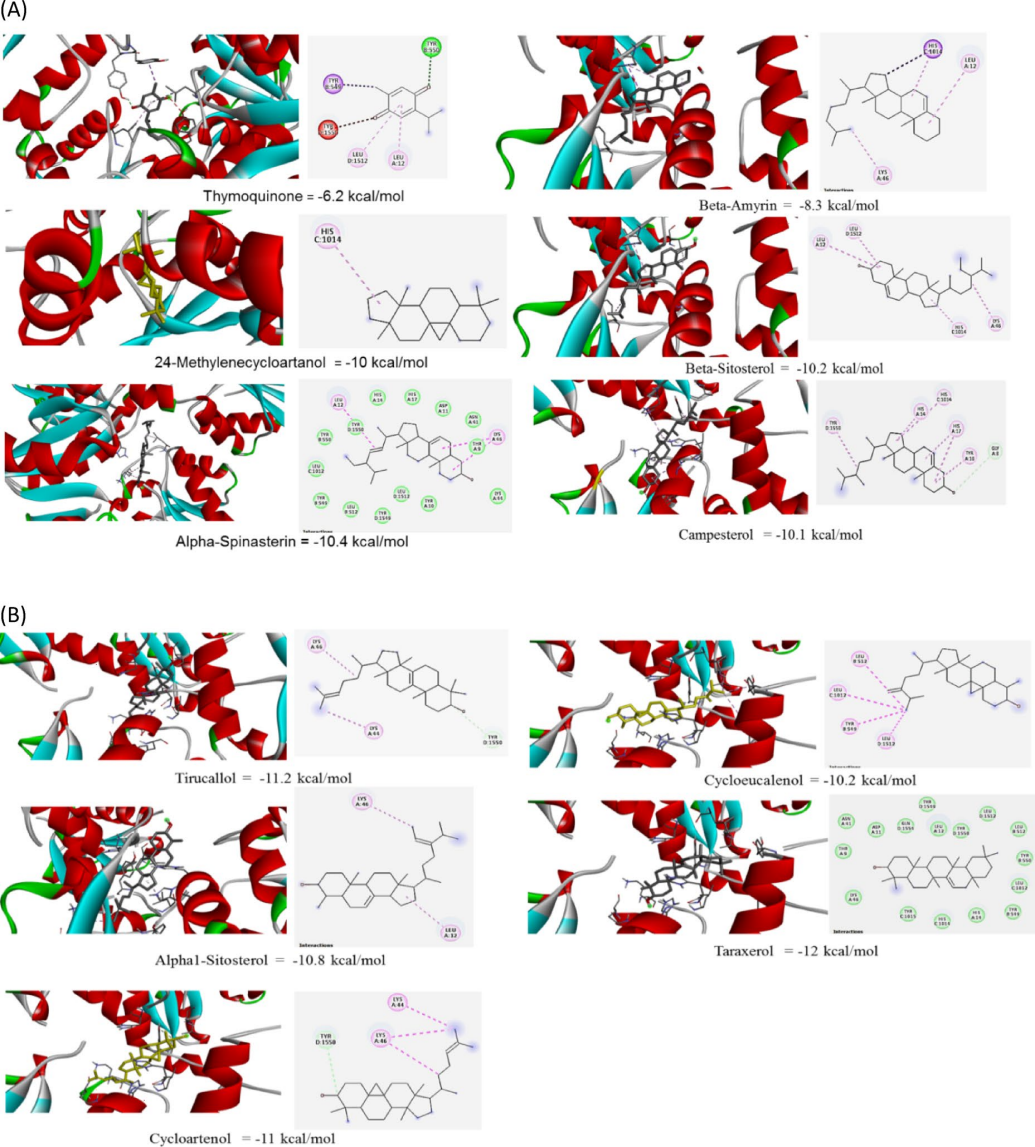
(**A**) Three-dimensional representation and 2D amino acid stick model of glycerol-3-phosphate cytidylyltransferases bonding six with *N. sativa* active components: Key colors in 2D-dimension representation: light blue: Van der Waals, green: conventional hydrogen bonding, orange: Hydrophobic interactions, pink: Pi-Pi stacked, red: unfavorable acceptor and violet: Pi-sigma. (**B**) Three-dimension representation and 2D stick model of amino acids of glycerol-3-phosphate cytidylyltransferases bonding with *N. sativa* active components. Key colors in 2D-dimension representation: light blue: Van der Waals, green: conventional hydrogen bonding, orange: Hydrophobic interactions, pink: Pi-Pi stacked, red: unfavorable acceptor and violet: Pi-sigma.

Thymoquinone bound to glycerol-3-phosphate cytidylyltransferase (CTP) with a moderate binding affinity (−6.2 Kcal/mol). The key interactions include a conventional hydrogen bond with TYR550, an unfavorable acceptor-acceptor interaction with TYR1550, a pi-alkyl interaction with LEU12, a pi-sigma interaction with TYR549, and Van der Waals interactions with LEU1512 (Fig. 5A and Supplementary Table T3) The hydrogen bond with TYR550 is likely crucial for the inhibitory effect of thymoquinone, potentially disrupting substrate binding or catalytic residue orientation. The unfavorable interaction with TYR1550 might limit the binding affinity, but also suggests opportunities for improving ligand design. The hydrophobic interactions contributed to the overall binding stability. Overall, thymoquinone appeared to be a potential inhibitor of CTP. Campesterol and cycloartenol featured several pi-alkyl interactions along with a carbon-hydrogen bond, the latter potentially offering a small amount of stabilization (Fig. 5A,B and Supplementary Table T3). Cycloeucalenol and beta-sitosterol also displayed pi-alkyl interactions, which were likely keys to their enzyme-inhibiting action by possibly obstructing substrate entry, causing shape alterations, or disrupting the arrangement of catalytic amino acids. Additionally, the alkyl interactions observed in cycloeucalenol, beta-sitosterol, and alpha1-sitosterol contributed to the strong binding and stability of their complexes with the enzyme. For 24-methylene-cycloartanol, pi-alkyl interactions were critical to its inhibitory effect on CTP. Regarding the enzymes targeted by alpha-spinasterol and taraxerol (Gat-D), and cycloeucalenol (CTP), these inhibitors mainly interacted through widespread Van der Waals forces and substantial hydrophobic interactions with many amino acids within the enzyme’s binding site. Their strong attraction and apparent occupation of an area crucial for substrate binding suggest a competitive mode of inhibition.

## Discussion

Plasmids are key vectors for antibiotic resistance gene transfer among bacteria. Plasmid curing demonstrates gene exchange between related strains and highlights the need for prudent antibiotic use. Natural plasmid elimination aids in distinguishing chromosomal from plasmid-borne genes benefiting both research and clinical applications^13,31^. The lack of plant-based plasmid-curing agents limits current therapies. Developing such agents to target plasmid-borne resistance in MDR bacteria is vital for advancing microbial genetics and treating chronic infections^32^. Plasmid-curing agents can reverse traits like antibiotic resistance and virulence. NSO and TQ are promising due to their safe and diverse medical benefits^33^. We hypothesized that NSO and TQ treatment would cure plasmids in *S. aureus* due to: (i) NSO induced cell wall damage^34^, (ii) plasmid replication disruption in wall-deficient protoplasts^35^ and (iii) NSO’s inhibition of plasmid conjugation loss^17^. This study analyzed cured plasmids via gel electrophoresis and assessed antibiotic resistance in treated *S. aureus* variants. Eleven *N. sativa* compounds were computationally screened (docking study) for their ability to inhibit CTP and Gat-D, key enzymes in cell wall biosynthesis. This is the first study reported plasmid-curing activity of *N. sativa.* Exposure of MDR *S. aureus* to NSO and TQ reversed plasmid-borne antibiotic resistance producing NSO-PRV and TQ-PRV variants. Isolates harbored 2–5 plasmids (6.48–51.36 kb), which is consistent with a previous finding of 3–5 plasmids ranging from 2.8 to 60 kb^36^. Our findings align with Udo et al.^37^, who observed 3–5 plasmids sized 2.8–38.0 kb. After NSO exposure, NSO-RPV and TQ-RPV variants formed small, non-pigmented colonies. Once grown without antibiotics, they reverted to typical *S. aureus* characteristics, indicating successful plasmid curing. Our findings align with earlier reports revealing that *S. aureus* forms slow-growing, non-hemolytic small colony variants after gentamycin exposure^38^. While extract *Dioscorea bulbifera*^39^ cured plasmids in Gram-negative bacteria at high MICs (50–1000 µg/mL), NSO and TQ achieved complete plasmid loss in all tested isolates at much lower MICs (4–7 µg/mL). Antibiotics that inhibit cell wall synthesis can induce *S. aureus* L-forms and lead to plasmid loss (3.4–28.2 kb) due to disrupted envelope-DNA interactions^40,41^. A previous study found 56.3% of resistance genes on both plasmids and chromosome with 33% showing horizontal transfer^42^. This study successfully eliminated most plasmid-borne resistance markers (6.48–51.38 kb). TQ showed plasmid-curing effects at MICs below its reported blood C-max (3.48 µg/mL)^43^ with plasmid loss even at 0.5 MIC. Combining TQ with other antibiotics enhanced plasmid elimination without inducing resistance consistent with a prior report on TQ/doxycycline and TQ/ofloxacin combinations^44^. In this study plasmid curing was 89.4% (17/19 plasmids) in *S. aureus* after using 0.5× MIC TQ plus 5% SDS While CRISPR-Cas achieved 96 ± 4% plasmid curing^45^. Treated isolates reverted to normal when grown without antibiotics, a trait beneficial for research and clinical use. In the present work, TQ, a bioactive compound from *Nigella sativa*, demonstrated a moderate binding with Gat-D and CTP, Comparatively, known inhibitors of Gat-D including Triciribine (–8.3 kcal/mol), Iodo tubercidin (–8.1 kcal/mol) and Sangivamycin (–8.0 kcal/mol) exhibit stronger binding affinities as reported by Kumar et al.^46^. These compounds interact with key residues in the enzyme’s active site forming multiple hydrogen bonds and hydrophobic contacts, thereby enhancing their binding strength. Although TQ’s binding energy is slightly weaker, its ability to form multiple stabilizing interactions and its known bioavailability suggest that it could exert biologically relevant inhibition at achievable concentrations. The non-competitive inhibition mechanism proposed for TQ further supports its potential to disrupt Gat-D function, potentially impairing peptidoglycan biosynthesis and contributing to plasmid loss in bacteria under stress. These findings warrant further in vitro validation to assess TQ’s therapeutic potential as a novel antimicrobial agent^46^. Compared to known inhibitor of CTPs as benzimidazole-phenyl-succinimide derivatives, which show half maximal inhibitory concentration (IC₅₀) values between 1 and 15 µM^47^, TQ’s binding energy suggests potential biological relevance at achievable concentrations. Other *N. sativa* components like campesterol, cycloartenol, and beta-sitosterol also demonstrated stabilizing hydrophobic interactions indicating competitive inhibition. Inhibition of CTP disrupts CDP-glycerol synthesis, a precursor for teichoic acid in bacterial cell walls. This disruption compromises cell wall integrity triggering stress responses that impair plasmid replication and segregation. Such stress-induced plasmid loss has been documented in bacterial systems^48^. The cumulative evidence supports the role of TQ and related compounds as biologically significant inhibitors of CTP with implications for antimicrobial strategies targeting plasmid-mediated resistance. Managing antibiotic resistance with *N. sativa* compounds is promising due to their dual action inducing plasmid loss and inhibiting conjugation^17^. Their potential prophylactic use before surgery offers a safer alternative to agents like SDS, which pose risks such as SDS colitis^49^. Limitations of this study include reliance on in vitro docking data and the absence of transcriptomic analysis. While docking suggests strong binding, actual biological activity requires validation in cellular models. The lack of transcriptomic profiling limits understanding of downstream gene expression changes. However, the observed reversion of antibiotic-resistant phenotypes to sensitivity after plasmid curing implies transcriptional alterations supporting the need for future transcriptomic studies. Future directions include in vivo validation, structure-activity relationship optimization and comprehensive omics-based profiling to elucidate the molecular impact of these inhibitors.

## Materials and methods

### Ethical approval

All procedures in this study were approved by the Institutional Animal Care and Use Committee of the Faculty of Veterinary Medicine, Zagazig University, Egypt (VETCU-IACUC). In addition, written informed consent was obtained from all human participants prior to their involvement in the study. Ethical approval for the use of human subjects was granted by the National Research Centre, Giza, Egypt. We confirm that all methods were performed in accordance with the guidelines and regulations of VETCU-IACUC and National Research Centre.

### *Nigella sativa* essential oil and thymoquinone

Essential oil derived from the seeds of *N. sativa* was obtained from Haraz Company in Egypt. Thymoquinone (product number 274666) was sourced from Sigma Aldrich, USA. *Nigella sativa* essential oil was prepared in a 10% DMSO solvent. Thymoquinone was initially dissolved in a 10% DMSO solution, and then further diluted with a 1:1 ethanol-water mixture to achieve the required concentration.

### Isolation and identification of *S. aureus* isolates

Twenty samples were collected from pus associated with human skin pyogenic infections (PU, No. = 9) and human urine (UR, No. = 11) sourced from private clinical bacteriology laboratories. Additionally, milk samples from cows (MK, No. = 9) were obtained from various dairy farms in Zagazig city, Sharkia governorate, Egypt. All samples were collected over a three-month period starting from January 2023. The samples were inoculated for 24 h at 37 °C on mannitol salt agar plates. Suspected yellow colonies were further cultured on sheep blood agar for an additional 24 h at 37 °C. All preliminary identified *S. aureus* isolates underwent genotypic characterization via PCR amplification of species-specific *nuc* gene. Following the manufacturer’s instructions, the molecularly identified *S. aureus* isolates were examined using Blue Staph Latex Kits (Pro-Lab Diagnostics) to assess clumping factor and protein A. The *S. aureus* strains were then preserved at −80 °C in a 10% glycerol solution prior to further use.

### Susceptibility of *S. aureus* isolates to antimicrobials and thymoquinone

Disc diffusion technique was used to assess the susceptibility of isolated *S. aureus* strains to antimicrobials and TQ^50^. A total of sixteen antimicrobial discs were used to determine antimicrobial susceptibility, including ampicillin (AMP), lincomycin (L), cefepime (FEP), cefotaxime (CTX), azithromycin (AZM), clindamycin (DA), amoxycillin/clavulanic acid (AMC), trimethoprim/sulphamethoxothazole (SXT), tobramycin (TOB), chloramphenicol (C), gentamicin (CN), tazobactam (TPZ), doxycycline (DO), ampicillin/sulbactam (SAM), linezolid (LZD), and ofloxacin (OFX). Briefly, a suspension containing 10^8^ CFU (colony forming units/mL) of freshly isolated bacteria was swabbed onto Muller Hinton agar (MHA) plates, onto which the antibiotic discs were placed For the purpose of identifying TQ susceptibility, a sterile 6 mm Whatman No.1 disc impregnated with TQ (25 µg) was affixed to the surface of the inoculated medium. At 37 °C, the plates were incubated for 24 h, after which the diameters of the inhibition zones were measured.

### Minimal inhibitory concentration of thymoquinone and antimicrobials

The minimal inhibitory concentration (MIC) of selected antimicrobials, and TQ against *S. aureus* strains was assessed using microtiter plates, following the guidelines established by CLSI in 2018^51^. In brief, TQ was initially dissolved in 10% DMSO, while tested antimicrobials were diluted in 100 µL of Muller Hinton broth. Each well was then filled with an equal volume of bacterial suspension containing 10^6^ CFU/mL. At 37 °C, the plates were incubated for 24 h After sealing. The absence of turbidity in the final dilution of TQ allowed for the recording of its MIC against the isolate. In the curing experiments, antibiotics were applied at a concentration of 0.5XMIC.

### Isolation of *Nigella sativa* oil and/or antimicrobial-resistant variants derived from parent *S. aureus* isolates

The original multidrug-resistant *S. aureus* strains were exposed to NSO and/or antimicrobials on Mueller-Hinton agar (MHA). Colonies forming around the NSO disc within the inhibition zone, though limited in number, were identified as resistant phenotype variants (RPVs) derived from the parent strains. The RPV to NSO (NSO-RPV) were specifically selected from these parent strains. Likewise, variants exhibiting resistance to both NSO and antimicrobials were also identified^44^. In brief, a freshly isolated colony from each strain was inoculated into brain heart infusion medium and incubated for 24 h at 37° C. The resulting broth culture was used to prepare an inoculum suspension at a concentration of 10^8^ CFU/mL in phosphate-buffered saline, which was subsequently swabbed onto an MHA plate. A sterile 6 mm Whatman No.1 filter paper disc, impregnated with 20 µL of NSO, was placed on the dry surface of MHA medium. Following this, an NSO disc (20 µL) was applied to the MHA surface. For combination of NSO and antimicrobials, the NSO disc was removed after 30 min at room temperature, and an antimicrobial disc was positioned in the same location. Control discs for antibiotics were also included. At 37 °C, the plates were incubated for 24 h. Subsequently, the RPV to NSO and/or antimicrobials, along with the control non-treated parent strains were selected for the assessment of cured plasmids using agar gel electrophoresis and for the evaluation of the resistance phenotypes of the cured *S. aureus* strains.

### Isolation of thymoquinone and/or antimicrobial-resistant variants derived from parent *S. aureus* isolates

Antimicrobials and/or TQ at subinhibitory concentrations were administered to the identified MDR *S. aureus* strains in brain-heart infusion medium. The RPV to TQ (TQ-RPV) derived from the parent strains were selected. Likewise, RPV to subinhibitory concentrations of both TQ and antimicrobials were included. As controls, sodium dodecyl sulphate (SDS, 5%), and 0.5XMIC-TQ/SDS (5%) were used. Briefly, brain heart infusion test medium was prepared with 0.5XMIC-TQ/mL and 10^5^/CFU/mL from the propagation broth medium, followed by incubation for 24 h at 37° C ^44^ Likewise, SDS (5%) and sublethal concentrations of TQ in combination with SDS (5%) and the selected antimicrobials were introduced into additional test media. At 37 °C, the tubes were incubated for 48 h with constant shaking. Subsequently, bacterial sediments from the previous treatments alongside the non-treated parent strains were collected to profile the plasmids in agarose to assess the resistance phenotypes of the cured resistant variants.

### Plasmid extraction and profiling

Plasmids were isolated from the original non-treated parent isolates, isolates that developed resistance to NSO and TQ and control ones using the EasyPure Mini Plasmid Extraction Kit (Sigma, USA). The extracted plasmid DNA was then analyzed by horizontal agarose gel electrophoresis against a 100 Kb marker (Sigma, USA) employing a 0.7% agarose gel in Tris-borate buffer^52^.

### Assessment of antibiotic resistance traits in cured resistant variants

Disc diffusion method was used to study the cured RPV that emerged from exposure to NSO, TQ, SDS, and TQ/SDS, as well as those that developed after treatment with TQ/antimicrobials. Briefly, bacterial sediments from strains treated with TQ, SDS and TQ/antimicrobials or SDS were collected and swabbed onto MHA medium. A half Macfarland bacterial density suspension was prepared from NSO-RPV, and then swabbed onto MHA surface. Following the drying of the plates, antimicrobial discs to which the parent strains exhibited resistance were selected and affixed to the surface of the plates. At 37 °C, the plates were incubated for 24 h and the diameters of the inhibition zones surrounding each antibiotic disc were measured.

### Molecular docking

To evaluate the inhibitory efficacy of *N. sativa* main phytochemical components against two prospective targets involved in cell wall synthesis, a computer-based virtual screening approach was used^53^. *Nigella sativa* chemical components were selected from databases (https://phytochem.nal.usda.gov/phytochem/search/list), with selection criteria based on their structure-activity relationships and potential metabolic pathways. The macromlecule glycerol-3-phosphate cytidylyltransferase (PDB, ID: 2B7L) and glutamine amidotransferase (PDB, ID: 5N9M) were examined in silico with ligands derived from eleven phytoconstituents of *N. sativa*, including thymoquinone (CID: 10281), 24-Methylenecycloartanol (CID: 94204), alpha-spinasterol (CID: 5281331), beta-amyrin (CID: 73145), beta sitosterol (CID: 222284), campesterol (CID: 173183), trucallol (CID: 101257), alpha1-sitosterol (CID: 9548595), cycloartenol (CID: 92110), cycloeucalenol (CID: 101690), and taraxerol (CID: 92097). The three-dimensional structures of CIP and Gat-D were obtained from the Protein Data Bank (PDB). The Autodock MGL tools were utilized for preparing the macromolecules and ligands and setting the grid box dimensions. Autodock Vina 1.2.0 and BIOVIA Discovery Visualization 2024 client softwares were employed for docking simulations between ligands and enzymes.

### Statistical analysis

The data were analyzed using SPSS version 26 (IBM Corp, Armonk, NY, USA). The Chi-square test was used to analyze categorical data including the differences in the antimicrobial resistance patterns of the recovered isolates from various sources. Additionally, one-way ANOVA and Tukey’s post hoc test were used to evaluate the effectiveness of plasmid curing in resistant phenotype variants of *S. aureus* treated with *N. sativa* essential oil and/or antimicrobials and TQ and/or antimicrobials. The normality and homogeneity among the treatment groups were determined utilizing Shapiro–Wilk’s and Levene’s tests, respectively. All experimental procedures were done in triplicat, and the results were expressed as mean ± standard error of the mean (SEM). The *p*-values were considered statistically significant if they were less than 0.05. Graphs were generated by GraphPad Prism version 8 (San Diego, CA, USA), and R-software version 4.4.3 (https://www.r-project.org/) using ggplot, pheatmap, and factoextra packages.

## Conclusion

*N. sativa* essential oil and TQ exhibited bactericidal effects on *S. aureus* with effective elimination of plasmids associated with resistant phenotypes originating from the treated parent strains. The cured resistant strains subsequently regained sensitivity to one or more antimicrobials to which the parent strain was previously resistant. Ligands of *N. sativa* components exhibited moderate to high binding affinities with the CTP and Gat-D cell wall enzymes, potentially disrupting their enzymatic activity by hindering substrate access to the active sites. These *N. sativa* active components may serve as plasmid curing agents in the field of microbial genetics, thereby helping to restrict the dissemination of antibiotic-resistance antibiotic-resistant genes from pathogenic *S. aureus*.

## Supplementary Information

Below is the link to the electronic supplementary material.

Supplementary Material 1

## Data Availability

The datasets generated and/or analyzed during the current study are available within the published article. Further information can be obtained from the corresponding author upon request.
