# Supplementary material for: Novel plasmid curing mediated restoration of antimicrobial sensitivity by Nigella sativa extract against multidrug resistant Staphylococcus aureus
Source: Sci Rep. 2026 Jan 18;16:2611. doi: 10.1038/s41598-025-33667-3 (PMC12820097; doi:10.1038/s41598-025-33667-3)
Supplement: Supplementary file 1 — Supplementary Material 1 [file 41598_2025_33667_MOESM1_ESM.docx]

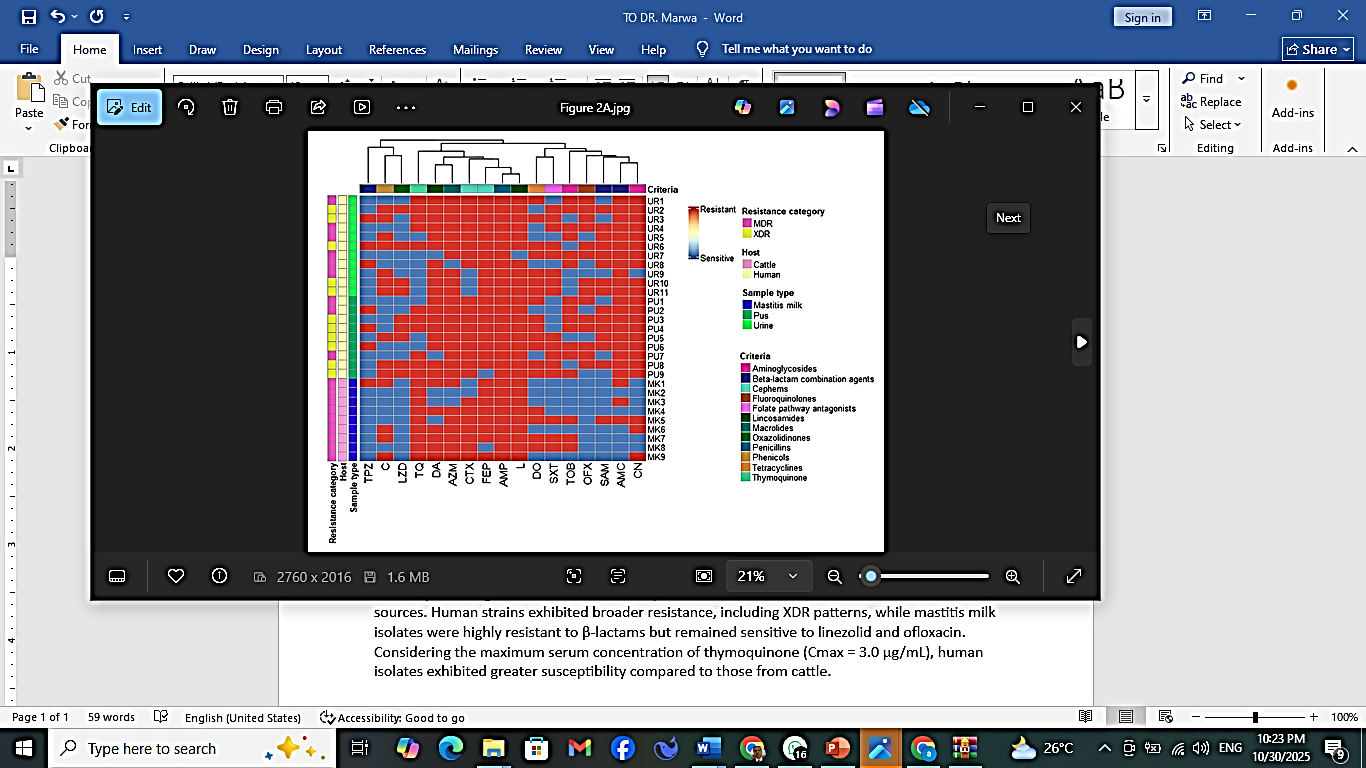


**Supplementary Figure S1.** Hierarchical clustering (HA) heatmap (A), and HA dendrogram (B) revealing the overall distribution of the tested *Staphylococcus aureus* isolates based on the phenotypic antimicrobial resistance patterns.

Different host, sample types, resistance categories, and antimicrobial classes are color-coded on the right of the heatmap. UR: human urine, PU: human pus, MK: mastitis milk, AMC: amoxycillin/clavulanic acid, AMP: ampicillin, FEP: cefepime, L: lincomycin, AZM: azithromycin, CN: gentamicin, CTX: cefotaxime, DA: clindamycin, TOB: tobramycin, SAM: ampicillin/sulbactam, OFX: ofloxacin, DO: doxycycline, SXT: trimethoprim/sulphamethoxazole, C: chloramphenicol, LZD: linezolid, TPZ: tazobactam, TQ: Thymoquinone, MDR: multidrug resistance, and XDR: extensively drug resistance.

**Supplementary Table T1.** Antimicrobial susceptibility and molecular weight of plasmids of resistant phenotypic variants cured with *N. sativa* oil and/or antimicrobials unlike the non-treated original parent *S. aureus* isolates

| ***S. aureus* isolates code No.** | **Molecular weights (kb) of plasmids of non-treated original parent isolates** | **Antimicrobial susceptibility and molecular weights of plasmids of resistant phenotypic** **variants cured with NSO and/or antimicrobials** | | | | | | |
| --- | --- | --- | --- | --- | --- | --- | --- | --- |
|  |  | **NSO** | | **NSO/antimicrobial combination** | | | | |
|  |  | **Plasmid molecular weight (kb)** | **MDR reversal** | **SXT** | **OFX** | **AZM** | **DA** | **DO** |
| **MK4** | 9.23,  44.29,  48.02 | 9.23,  44.29,  48.02 | DO (28),  AZM (20)  FEP (26) | 9.23 | 9.23 | 9.23 | 48.2 | 9.23,  44.29, 48.02 |
| **MK6** | 51.36,  44.29,  28.16,  6.48 | 51.36,  6.48 | AZM (20) | 51.36  6.48 | 51.36  6.48 | 51.36, 44.29, 28.16  6.48 | 51.36 | 51.36, 28.18,  6.48 |
| **UR1** | 12.06,  48.02 | 12.6 | CN (24)  SXT (29) | 12.6 | 12.06, 48.02 | 12.6 | 12.06,  48.02 | 12.06,  48.02 |
| **UR9** | 51.36,  44.29,  9.23 | 51.36,  44.29,  9.23 | SXT (36)  AZM (25) | 51.36,  44.29,  9.23 | 51.36,  44.29,  9.23 | 51.36,  44.29,  9.23 | 51.36,  44.29,  9.23 | 51.36,  44.29,  9.23 |
| **PU1** | 44.29,  9.23 | 44.29 | DO (28)  OFX (22)  DA (22) | 44.29,  9.23 | 44.29 | 44.29 | 44.29 | 44.29,  9.23 |
| **PU9** | 44.29,  41.88,  9.23,  7.51,  6.48 | 7.51,  6.48 | AZM (32)  DO (20)  CN (29) | 44.29, 41.88, 7.51,  6.48 | 44.29,  41.88,  9.23,  7.51,  6.48 | 44.29,  41.88,  9.23,  7.51,  6.48 | 44.29,  7.51  6.48 | 44.29,  41.88,  7.51,  6.48 |

*S. aureus*: *Staphylococcus aureus*, MK: milk, UR: urine, PU: pus, NSO: *N. sativa* oil, kb: kilobase pairs, SXT: trimethoprim/ sulpha-methoxazol, OFX: ofloxacin, AZM: azithromycin, DO: doxycycline, DA: clindamycin, FEP: cefepime, CN: gentamicin.

**Supplementary Table T2.** Cured plasmids of resistant phenotypic variants to 0.5X MIC-TQ, 0.5X MIC-TQ plus antibiotics, SDS, and 0.5X MIC-TQ plus SDS compared with non-treated original parent S. aureus isolates.

| *S. aureus* isolates code No. | Molecular weights (kb) of plasmids of non-treated original parent isolates | Antimicrobial susceptibility and molecular weights of plasmids of resistant phenotypic variants cured with TQ and/or antimicrobials and SDS and/or TQ | | | | | | | | |
| --- | --- | --- | --- | --- | --- | --- | --- | --- | --- | --- |
|  |  | **0.5X MIC of TQ** | | **0.5X MIC of TQ/antimicrobial** | | | **SDS** | | **SDS/ 0.5X MIC of TQ** | |
|  |  |  |  | **DO** | **DO+CN** | |  |  |  |  |
|  |  | **Plasmid molecular weight (kb)** | **MDR reversal** | **Plasmid molecular weight (kb)** | **Plasmid molecular weight (kb)** | **MDR reversal** | **Plasmid molecular weight (kb)** | **MDR reversal** | **Plasmid molecular weight (kb)** | **MDR reversal** |
| **MK4** | 9.23,  44.29  48.02 | Nil | Nil | 44.29  48.02 | 9.23,  44.29  48.02 | NBG | 9.23 | AZM (25)  DO (24) | 48.02 | FEP (23) |
| **MK6** | 51.36, 44.29,  28.16  6.48 | 51.36,  6.48 | AZM (25) | 51.36,  28.16  6.48 | 51.36,  28.16  6.48 | NBG | 51.36,  28.16  6.48 | AZM (26)  DO (20) | 51.36, 44.29,  28.16  6.48 | AZM (26)  DO (20) |
| **UR1** | 12.06,  48.02 | 12.06 | TOB (28)  CN (29) | 12.06,  48.02 | 12.06,  48.02 | ND | 48.2 | CN (16) | 12.06,  48.02 | CN (20)  TOB (28)  AZM (18) |
| **UR9** | 51.36, 44.29,  9.23 | 51.36,  9.23 | AZM (26) | 51.36, 9.23 | 51.36, 44.29,  9.23 | AZM (22) | 51.36, 44.29,  9.23 | Nil | 51.36, 44.29,  9.23 | AZM (25) |
| **PU1** | 44.29,  9.23 | 44.29,  9.23 | DO (28)  OFX (21)  DA (21)  CN (20) | 44.29,  9.23 | 44.29,  9.23 | DO (28)  OFX (22)  DA (22) | 44.29,  9.23 | DO (19)  TOB (15) | 44.29,  9.23 | DO (20)  OFX (35)  AZM (19)  CN (27) |
| **PU9** | 44.29, 41.88, 9.23,  7.51,  6.48 | 44.29 | AZM (21)  DO (18)  DA (21) | 44.29,  7.51,  6.48 | 44.29,  9.23,  7.51,  6.48 | AZM (25)  DO (21)  DA (23)  L (20) | 7.51,  6.48 | CN (19)  DO (16) | 44.29,  9.23 | DO (19)  CN (22)  AZM (25) |

*S. aureus*: *Staphylococcus aureus*, MK: milk, UR: urine, PU: pus, TQ: thymoquinone, SDS: sodium dodecyl sulfate, MIC: minimum inhibitory concentration, DO: doxycycline, CN: gentamicin, kb: kilobase pairs, AZM: azithromycin, TOB: tobramycin, CN: gentamicin, OFX: ofloxacin, DA: clindamycin, L: lincomycin, FEP: cefepime, NBG: no bacterial growth, ND: not done.

**Supplementary Table T3.** Interactions of eleven *Nigella sativa* components with specific amino acids within two *S. aureus* cell wall synthesis enzymes (Glycerol-3-phosphate cytidylyltransferase and glutamine amidotransferase.

| **Enzyme** | **Key interaction** | **Thymoquinone** | **24-Meth-ylene cycloartanol** | **Beta-**  **sitosterol** | **Cycloeucalenol** | **Camp sterol** | **Alpha**  **Sito-sterol** | | **Cycloartenol** | **TriuCalol** | **Alpha**  **spina-sterol** | **Taraxerol** | **Beta-armine** |
| --- | --- | --- | --- | --- | --- | --- | --- | --- | --- | --- | --- | --- | --- |
| **Glycerol-3-phosphate cytidylyltransferase** | **Binding affinity(kcal/mol)** | -6.2 | -10 | -10.2 | -10.2 | -10.1 | -10.8 | | -11 | -11.2 | -10.4 | -9.6 | -8.3 |
|  | **Conventional hydrogen bonding** | TYR550 | Nil | Nil | Nil | Nil | Nil | | Nil | Nil | Nil | Nil | Nil |
|  | **Unfavorable acceptor interaction** | TYR1550 | Nil | Nil | Nil | Nil | Nil | | Nil | Nil | Nil | Nil | Nil |
|  | **Pi alkyl**  **interaction** | TYR  12 | HIS1014 | HIS1014 | LEU512,  1012,  1512 | 5AA | Nil | | Nil | Nil | Nil | Nil | LYS46 |
|  | **Pi Sigma interactions** | TYR 549 | Nil | Nil | Nil | Nil | Nil | | Nil | Nil | Nil | Nil | HIS 1014 |
|  | **Van der Waals** | LEU 1512 | Nil | Nil | Nil | Nil | Nil | | Nil | Nil | THR 9 | 16AA | 16AA |
|  | **Alkyl interaction** | Nil | Nil | LEU1512, 12,  LYS16 | TYR549 | LEU12,  LYS 46 | LYS  46,44 | | LYS46 | LYS46,  LEU12 | Nil | Nil | Lys 12 |
|  | **Carbon hydrogen bond** | Nil | Nil | Nil | GLY8 | Nil | TYR 1550 | TYR 1550 | | Nil | Nil | Nil | Nil |
| **Glutamine**  **amidotransferase** | **Binding affinity(K/caL)** | -6.4 | -9.0 | -9.5 | -9.7 | -9.0 | -10.1 | | -9.8 | -9.7 | -9.7 | -9.6 | -7.7 |
|  | **Conventional hydrogen**  **bonding** | 5AA | Nil | Nil | Nil | Nil | Nil | | Nil | Nil | Nil | Nil | Nil |
|  | **Van der Waals** | TYR187, 17,  CYS 194 | VAL40 | VAL40,  PRO 191,  ILE24 | 18AA | VAL40,  PRO 191,  ILE24 | PRO79 | | PRO79 | Nil | ARG128,  TYR17,  PRO79 | PRO  107 | PRO  79 |
